# Enhanced Phytoremediation of Bisphenol A in Polluted Lake Water by Seedlings of *Ceratophyllum demersum* and *Myriophyllum spicatum* from In Vitro Culture

**DOI:** 10.3390/ijerph18020810

**Published:** 2021-01-19

**Authors:** Chong Zhao, Guosen Zhang, Jinhui Jiang

**Affiliations:** 1 School of Life Sciences, Central China Normal University, No. 152, Luoyu Avenue, HongShan District, Wuhan 430079, China; zhaochong426@mail.ccnu.edu.cn (C.Z.); zhangguosen1989@foxmail.com (G.Z.); 2 The College of Urban & Environmental Sciences, Central China Normal University, No. 152, Luoyu Avenue, HongShan District, Wuhan 430079, China

**Keywords:** submerged macrophytes, indigenous microorganisms, phytoremediation, Bisphenol A, in vitro culture

## Abstract

Bisphenol A (BPA) is a typical endocrine disruptor that causes problems in waters all around the world. In this study, the effects of submerged macrophytes (*Ceratophyllum demersum* and *Myriophyllum spicatum*) cultured in vitro on the removal of BPA at two initial concentrations (0.5 mg L^−1^ vs. 5.0 mg L^−1^) from Donghu lake water were investigated, using different biomass densities (2 g L^−1^ vs. 10 g L^−1^) under different nutrient conditions (1.85 mg L^−1^ and 0.039 mg L^−1^ vs. 8.04 mg L^−1^ and 0.175 mg L^−1^ of the total nitrogen and phosphorus concentration, respectively), together with the effect of indigenous microorganisms in the water. The results showed that indigenous microorganisms had limited capacity for BPA removal, especially at higher BPA initial concentration when its removal rate amounted to about 12% in 12 days. Addition with plant seedlings (5 cm in length) greatly enhanced the BPA removal, which reached 100% and over 50% at low and high BPA initial concentration in 3 days, respectively. Higher biomass density greatly favored the process, resulting in 100% of BPA removal at high BPA initial concentration in 3 days. However, increases in nutrient availability had little effect on the BPA removal by plants. BPA at 10.0 mg L^−1^ significantly inhibited the growth of *M. spicatum*. Therefore, *C. demersum* may be a candidate for phytoremediation due to greater efficiency for BPA removal and tolerance to BPA pollution. Overall, seedlings of submerged macrophytes from in vitro culture showed great potential for use in phytoremediation of BPA in natural waters, especially *C. demersum*.

## 1. Introduction

Bisphenol A (BPA) has received a great deal of attention as a typical endocrine disrupting chemical in recent years [1,2]. Because of its widespread usage, this compound has frequently been detected in all kinds of environmental media, including air, soil, sediment and especially water [1,3]. Sewage treatment plants and urban and agricultural runoff are usually the sources of such pollution [2,4]. Low concentrations of BPA ranging from ng L^−1^ to μg L^−1^ have been reported in different aquatic systems. For example, Salgueiro-González [5] found average levels of BPA of 0.054–0.103 μg L^−1^ in three estuaries in Spain, while Gorga [6] found maximum concentrations of 0.084–0.229 μg L^−1^ in test rivers in Spain, and Jonkers [7] found levels as high as 0.683 μg L^−1^ in rivers of Portugal. In China, Xiong [8] reported BPA levels in water samples from Beijiang river of 0.06–0.72 ng L^−1^. However, in Donghu lake in Wuhan, which is the largest urban lake in China, the concentration of BPA in the water was 20–534 ng L^−1^, which was attributed to the impact of domestic sewage [9]. Electronic waste recycling sites and waste landfill leachates have been found to contain BPA at levels as high as 860 μg L^−1^ and 17.2 mg L^−1^, respectively [8,10]. According to early reports, BPA can disturb the behavior and development of many species by interference with synthesis, secretion, transport, binding, action, or elimination of natural hormones in the body, even when present at levels as low as 1 μg L^−1^ [1,11]. Moreover, it possesses genotoxic and carcinogenic properties and induces oxidative stress [1]. Therefore, there is an urgent need to remove such contaminants from aquatic environments.

Microorganisms such as bacteria have been shown to have the ability to degrade BPA [12]. These BPA-degrading bacteria are believed to be ubiquitous in aquatic environments, resulting in widespread BPA biodegradation in such systems [13]. However, most of these organisms are limited in their ability to degrade BPA in natural ecosystems, although some strains of *Sphingomonas* and *Pseudomonas* isolated from severely polluted environments showed high efficiency in simulating experiments [14]. Because lake water is normally slightly contaminated by BPA, it is reasonable to assume that indigenous bacteria in waters such as Donghu lake at Wuhan would also show limited BPA degradation ability. It should also be noted that the entire bacterial community, not just the isolated BPA degraders, is responsible for the biodegradation process in actual ecosystems [15]. Accordingly, changes in microbial community structure will affect the BPA biodegradation efficiency [16], making it necessary to verify the effects of the indigenous microbial community on BPA removal from natural lake water.

Phytoremediation is considered as an eco-friendly and low-cost method to remove pollutants from ecosystems, especially in situ [17]. Nevertheless, its efficiency can vary greatly among plant species [18]; therefore, suitable plant species should be selected for remediation activities. Although some algal and herbaceous species have been shown to have the potential to remove BPA from aqueous solutions [18,19], their intrinsic properties have inhibited their application. However, submerged macrophytes are considered to be great candidates for phytoremediation of organic pollutants from water. For example, *Potamogeton crispus* has been shown to have good ability in removing phthalic acid esters from water [20], while *Hydrilla verticillata*, *Potamogeton oxyphyllus* and *Ceratophyllum demersum* have been shown to have good capacity for BPA remediation [21]. Additionally, our early research showed that submerged plants including *C. demersum* and *Myriophyllum spicatum* had good BPA removal ability, indicating their potential for application in BPA phytoremediation [22].

Submerged vegetation has declined greatly in recent years [23], leading to a shortage of plant materials used in restoration programs. Therefore, plant materials collected using in vitro culture methods are preferred for restoration activities because of their rapid propagation, easy collection and high success in transplantation [24]. However, no studies have investigated BPA removal by such plants to date. Our early experimental results showed that plant materials from in vitro culture based on tissue culture methods were better at BPA removal than natural plants under the same conditions, suggesting they will perform better in phytoremediation activities (data were shown in Supplementary materials). Therefore, it is interesting to investigate their BPA removal ability under different conditions.

In this study, plant seedlings of *C. demersum* and *M. spicatum* from in vitro culture were tested for their ability to remove BPA from natural lake water under different biomass densities, nutrient levels (represented as TN and TP) and initial BPA concentrations, together with the effects of indigenous microorganisms in the water on BPA removal to simulate phytoremediation scenarios. Growth performance of these plants was also investigated in the presence of different levels of BPA in order to test their tolerance. Specifically, the following hypotheses were tested: (1) indigenous microorganisms have limited BPA removal ability; (2) addition of plant seedlings from tissue culture greatly favors BPA removal, although differences should be seen between plant species; and (3) plant seedlings have great tolerance to BPA.

## 2. Materials and Methods

### 2.1. Plant Materials

Aseptic seedlings of *C. demersum* and *M. spicatum* were obtained from in vitro aseptic culture after surface disinfection based on the procedure described by [24]. Shoot fragments of both plant species that were 2–3 cm in length were cleaned carefully in running tap water as explants for disinfection. The disinfection process consisted of submersion in 70% ethanol for 30 s followed by 10% commercial bleach (5.75% sodium hypochlorite by weight) for 2–3 min. Next, all fragments were immediately rinsed in sterilized deionized water five times, then cultured in Murashige and Skoo based liquid media (MS) plus 3% sucrose that had been autoclaved at 121 °C for 30 min. Every two weeks, the media was renewed to improve the growth of seedlings. After at least four generations, similar seedlings of each species at 5 cm in length were selected for experiments.

### 2.2. Reagent

Bisphenol A (purity ≥ 97%) was purchased from Acros Organics (Pittsburgh, PA, USA) and used to prepare a stock standard solution of 5 mg mL^−1^ BPA in methanol (HPLC grade, TEDIA, Fairfield, OH, USA) that was stored in the dark at 4 °C. Working solutions were prepared by dilution with lake water. Acetonitrile and trifluoroacetic acid used for HPLC analyses were of HPLC grade, while other chemicals were analytically pure which were purchased from Sinopharm Chemical Reagents (Shanghai, China).

### 2.3. Experimental Set-Up

Aseptic seedlings of *C. demersum* and *M. spicatum* were tested for their ability to remove BPA from Donghu lake water (collected at 30°32′59.15″ N, 114°25′29.53″ E) that had been spiked with BPA at initial concentrations of 0.5 mg L^−1^ or 5.0 mg L^−1^. Tests were conducted at biomass densities of 2 g L^−1^ and 10 g L^−1^. Groups without plants, but with indigenous microbes, as well as groups without biota (autoclaved lake water without plants) were used as controls. To investigate the removal of BPA from water in more severely eutrophic lakes, samples were amended with nutrients. Therefore, two parallel experiments were performed under the original nutrient condition and the nutrient addition condition. Specifically, the total nitrogen concentration and total phosphorus concentration were 1.85 mg L^−1^ and 0.039 mg L^−1^, respectively, for the original nutrient condition of lake water, and 8.04 mg L^−1^ and 0.175 mg L^−1^ for the nutrient addition condition. The added nutrients consisted of KH_2_PO_4_ and KNO_3_. The experimental duration was 12 days, at which time plants were harvested and oven dried at 80 °C to constant weight to determine the dry weight.

To test the BPA tolerance of the aseptic seedlings of both species excluding the effects of indigenous microbes in lake water, autoclaved lake water spiked with 0.5 mg L^−1^, 5.0 mg L^−1^ and 10 mg L^−1^ of BPA was used to culture the plants. Plant growth with no BPA was used as a control. The initial biomass density was 10 g L^−1^. After 12 days of growth, plants were harvested and their dry weights were recorded.

All of the above experiments were conducted in sterile Erlenmeyer flasks containing 500 mL water covered with sterile polytetrafluoroethylene (PTFE) membranes. Experiments were conducted under four fluorescent lamps (3500 lx) with a light period of 16 h at 25 ± 2 °C. Three replicates were used for each treatment.

### 2.4. Determination of Residual BPA in Water

Water samples (4 mL) were taken to determine the residual BPA every 3 days. After being filtered through 0.7 μm GF membranes, the residual BPA concentration in water was measured using a reverse phase HPLC (LC20AT, Shimadzu, Kyoto, Japan) equipped with a UV detector at a wavelength of 278 nm using a C18 column (250 mm × 4.6 mm × 5 μm, Agilent, Santa Clara, CA, USA). The HPLC mobile phase was 65% acetonitrile and 35% ultrapure water containing 0.3% trifluoroacetic acid (*v*/*v*). The BPA retention time was 3.620 ± 0.005 min at a flow rate of 1 mL/min and the injection volume was 20 μL. The detection limit for BPA measurement was 0.0023 mg L^−1^.

### 2.5. Data Collection and Analysis

The BPA removal rate was calculated. The relative growth rate of plants (mg mg^−1^ d^−1^) was also calculated by the equation: relative growth rate = (lnW_t_ − lnW_0_)/t, where W_0_ (mg) and W_t_ (mg) indicated the dry weights of plants at the initial time (d) and t time (d).

Two-way ANOVA was performed to analyze the effects of initial BPA concentration and biota treatment (including no biota recorded as non-bio, indigenous microbes recorded as microbe, and plants and indigenous microbes recorded as plant + microbe) on BPA removal rate at each detecting time, and to analyze the effects of initial BPA concentration and biomass density on RGR for both species under both nutrient conditions. Three-way ANOVA was used to analyze the effects of plant species, biomass density, and initial BPA concentration on BPA removal under both nutrient conditions at each detecting time. When a significant interaction between factors was found, simple analysis was used for further steps. These analyses consisted of two-way ANOVA after a three-way ANOVA test, and/or independent samples T test between two factors or Bonferroni’s test as multiple comparisons among factors after a two-way ANOVA test. The natural distribution and homogeneity of variance of data were checked using the Kruskal-Wallis H test and Levene’s test, respectively. If data did not meet these conditions, they were transformed to meet the requirement. Statistical analysis was performed in SPSS v18.0 (IBM, Armonk, NY, USA). Statistical significance was defined at *p* < 0.05.

## 3. Results

### 3.1. BPA Removal Was Greatly Affected by Initial Concentration and Biota Treatment under Different Nutrient Conditions

**Figure 1:**
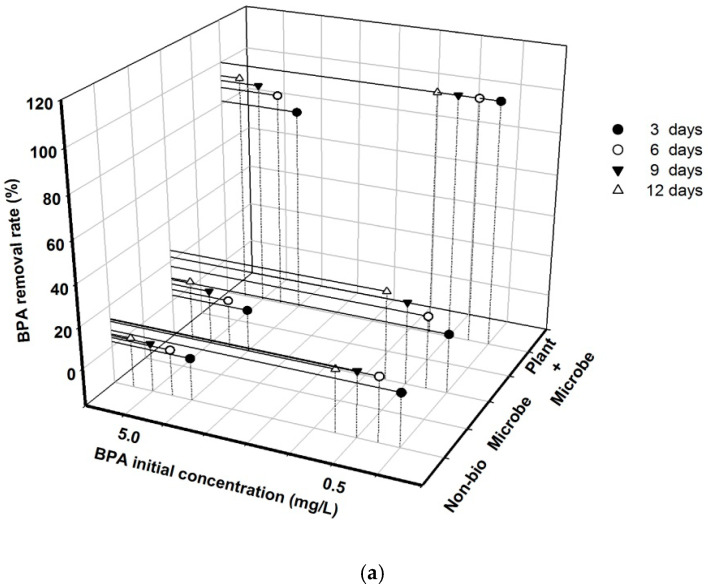

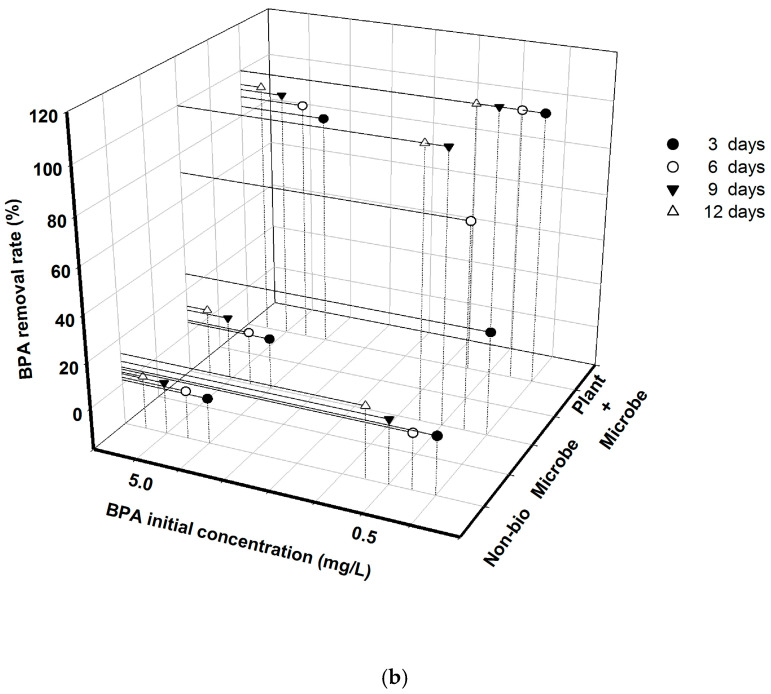
BPA (Bisphenol A) removal rate varied greatly with different initial concentration and biota treatment under original nutrient condition (**a**) and nutrient addition condition (**b**) over 12 days.

There were no significant interaction effects between initial BPA concentration and biota treatment on BPA removal rate under the original nutrient conditions during all experimental times (*p* > 0.05, Table 1). However, both biota treatment and initial BPA concentration exerted significant effects on BPA removal rate at all sampling days under such nutrient conditions (*p* < 0.05). Additionally, the BPA removal rate was always significantly smaller at high initial BPA concentration than at low initial BPA concentration (*p* < 0.05, Figure 1a). The plant + microbe treatment greatly increased BPA removal relative to the microbe treatment and the non-bio treatment at all times (*p* < 0.01), with removal rates of 100% and 81.3% being observed for initial concentrations of 0.5 mg L^−1^ and 5.0 mg L^−1^, respectively, at 3 days. The microbe treatment groups also had the ability to increase the BPA removal rate relative to the non-bio treatment groups, although they only showed significance on day 12 (*p* < 0.05), when 13.2% and 5.3% increases were observed for the 0.5 mg L^−1^ and 5.0 mg L^−1^ initial BPA concentration groups, respectively.

Under nutrient addition conditions, there was a significant interaction effect between the initial BPA concentration and biota treatment on BPA removal rate at all experimental days, except for day 3 (*p* < 0.05, Table 1). At low initial BPA concentration, both the microbe treatment and plant + microbe treatment significantly increased the BPA removal rate relative to the non-biota treatment at 6, 9 and 12 days (*p* < 0.01). However, there were no significant differences in BPA removal rates between the microbe and plant + microbe treatment groups after 9 days (*p* > 0.05). At high initial BPA concentration, the plant + microbe treatment groups had greater BPA removal rates than the others at 6, 9 and 12 days (*p* < 0.01). However, the microbe treatment did not differ significantly from that of the non-biota treatment (*p* > 0.05). In the non-biota treatment groups, the initial BPA concentration only had a significant effect on the BPA removal rate on day 12 (*p* < 0.05). In the microbe treatment groups, differences in initial BPA concentration resulted in significant variations in BPA removal rate (*p* < 0.05), which reached 100% in 9 days for the lower initial concentration, but was only 12.5% at 12 days for the higher initial concentration. In the plant + microbe treatment groups, the effects of initial BPA concentration on BPA removal rate were similar to those in the microbe treatment groups, except that the BPA removal rate was always >85%.

Evaluation of the plant + microbe and indigenous microbe groups revealed that the BPA removal rate was always significantly smaller at high initial BPA concentration than at low initial BPA concentration under both nutrient conditions (*p* < 0.05, Figure 1), except for that of the plant + microbe groups under nutrient addition conditions on day 12.

Nutrient addition greatly accelerated BPA removal by indigenous microbes at low initial BPA concentration (*p* < 0.05, Figure 1), while it showed no significant effect on BPA removal rate at high initial BPA concentration on day 12 (*p* > 0.05). Moreover, nutrient addition had no significant effect on BPA removal from the plant + microbe groups at both initial BPA concentrations at all sampling times (*p* > 0.05).

### 3.2. Species Type, Initial BPA Concentration and Biomass Density Affected BPA Removal in Plant + Microbe Groups under Different Nutrient Conditions

**Figure 2:**
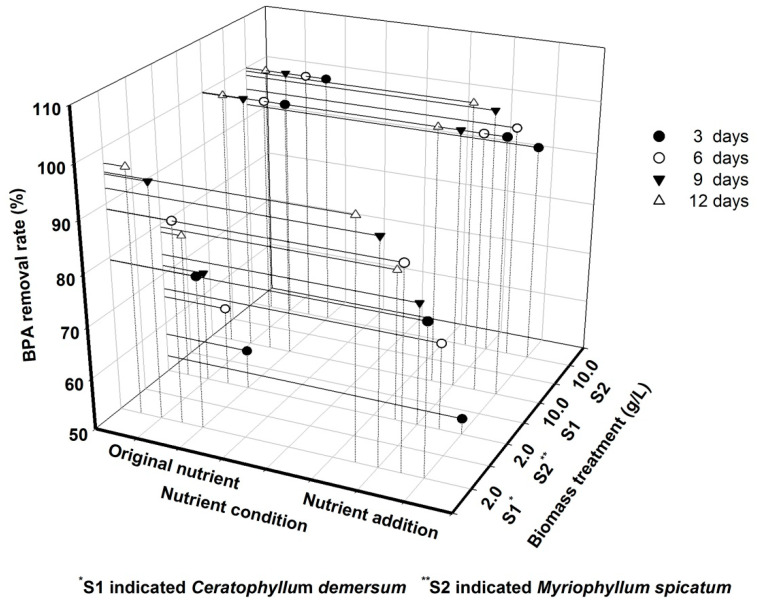
BPA removal rate for *Ceratophyllum demersum* and *Myriophyllum spicatum* and indigenous microbes with different biomass densities and different nutrient conditions at 5.0 mg L^−1^ initial BPA concentration.

Under both nutrient conditions, there were great interactions between factors including species type, initial BPA concentration and biomass density at all sampling times (Table 2, *p* < 0.01). Therefore, a simple analysis was conducted to evaluate the effects of these factors. At low initial BPA concentration under both nutrient conditions, the BPA removal rate was always 100% for all groups treated with plant + microbe at all testing times. Therefore, the BPA removal rate at high initial BPA concentration was analyzed in further steps (Figure 2). There were also significant interaction effects observed between species type and biomass density at high initial BPA concentration under both nutrient conditions at all times (*p* < 0.01), suggesting we should test the effects of species type in both biomass density groups and the effects of biomass density for both species.

When the biomass density was 2 g L^−1^ under the original nutrient conditions, *C. demersum* had a greater BPA removal rate than *M. spicatum* (*p* < 0.01, Figure 2). However, at 10 g L^−1^ biomass density under the original nutrient condition, there was no significance between the BPA removal rate of the two species (*p* > 0.05). Similar patterns of the effects of species type at different biomass densities were found under nutrient addition conditions (Figure 2).

Under both nutrient conditions, biomass density had a significant effect on BPA removal for both species (*p* < 0.05, Figure 2), with higher biomass density resulting in a greater BPA removal rate.

Nutrient addition generally showed no significant effect on BPA removal rate in plant + microbe groups (*p* > 0.05, Figure 2), although it slightly accelerated BPA removal for *M. spicatum* at a biomass density of 10 g L^−1^ and an initial BPA concentration of 5.0 mg L^−1^ (*p* > 0.05).

### 3.3. BPA Tolerance of Aseptic Seedlings of C. demersum and M. spicatum

Under the original nutrient conditions, there were significant interaction effects between initial BPA concentration and biomass density on RGR for both species (*p* < 0.05, Table 3). Under the nutrient addition condition, no significant interaction effects were found on RGR for *C. demersum* (*p* > 0.05), but there were significant effects observed for *M. spicatum* (*p* < 0.05). Therefore, a simple analysis was conducted.

**Figure 3:**
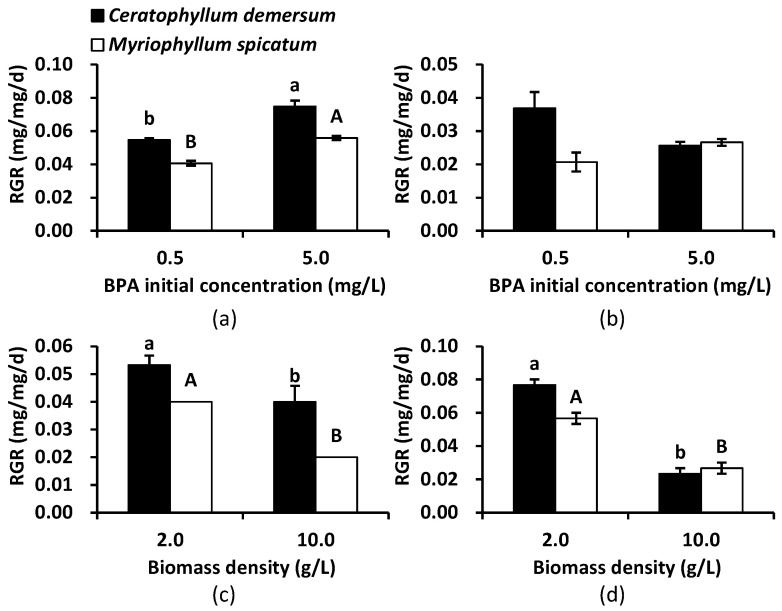
Relative growth rate (RGR) of *Ceratophyllum demersum* and *Myriophyllum spicatum* under original nutrient condition as affected by initial BPA concentration with a biomass density of 2 g L^−1^ (**a**) and 10 g L^−1^ (**b**) and as affected by biomass density with an initial BPA concentration of 0.5 mg L^−1^ (**c**) and 5.0 mg L^−1^ (**d**). Different letters (“a” and “b” or “A” and “B”) above columns indicate a significant difference at 0.05.

Evaluation of the RGR of both species under the original nutrient condition revealed that it significantly increased at high initial BPA concentration when the biomass density was 2.0 g L^−1^ (*p* < 0.05, Figure 3a), while it did not differ significantly between the two initial BPA concentrations when the biomass density was 10.0 g L^−1^ (*p* > 0.05, Figure 3b). At both initial BPA concentrations, biomass density significantly affected the RGR of both species (*p* < 0.05, Figure 3c,d), with a higher biomass density leading to a lower RGR. Generally, *C. demersum* showed better growth performance than *M. spicatum*.

**Figure 4:**
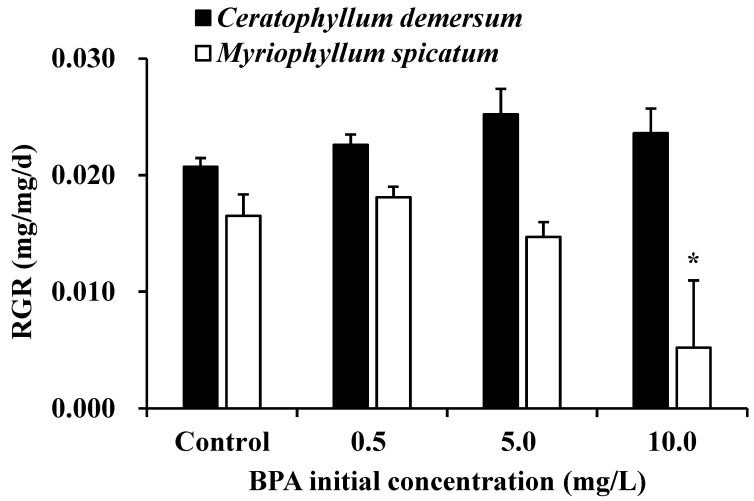
Relative growth rate (RGR) of aseptic seedlings of *Ceratophyllum demersum* and *Myriophyllum spicatum* under different BPA treatments without indigenous microbes. * indicates a significant difference at the 0.05 level.

The relative growth rate of aseptic seedlings of *C. demersum* did not change significantly under initial BPA concentrations of 0.5–10.0 mg L^−1^ (*p* > 0.05, Figure 4), although it was slightly larger at the high initial BPA concentration when no indigenous microbes were present. However, BPA showed growth inhibition of the aseptic seedlings of *M. spicatum*. At an initial BPA concentration of 10.0 mg L^−1^, the growth rate of *M. spicatum* decreased significantly comparing with other BPA treatments and the control (*p* < 0.05). Generally, aseptic seedlings of *C. demersum* grew better in BPA polluted water than *M. spicatum*.

## 4. Discussion

### 4.1. Indigenous Microbes Showed Limited Ability for BPA Removal

Indigenous microbes in Donghu lake water showed a limited BPA removal rate of about 10% in 12 days under present nutrient conditions (calculated based on the difference between microbe groups and non-bio groups). This removal rate was much lower than that in river water from three rivers in Japan in which the BPA removal rate was about 100% in 7 days [25]. Previous research has proven that BPA removal was shown to primarily occur via microbial degradation [26]. Thus, the low rate in Donghu lake water was probably because of the absence of highly-BPA-degrading microorganisms. According to early research, specific enzymes produced by bacteria, such as cytochrome P450 monooxygenase and laccase, may be responsible for these degradation processes [12]. These enzymes are commonly activated by substrates such as specific pollutants; however, in Donghu lake water, the BPA concentration was lower than 0.6 μg L^−1^, suggesting low BPA availability which was unfavorable for the activation of specific BPA degrading enzymes. As a result, highly-BPA-degrading bacteria may not be dominant under such conditions. Therefore, it was reasonable to find that bacteria with high BPA degradation ability was mostly isolated from severely polluted environments, such as landfill leachate [27].

Nutrient addition substantially enhanced BPA removal of indigenous microorganisms, with removal rates of 100% being observed in 9 days when the initial BPA concentration was 0.5 mg L^−1^. This was likely caused by improved development of microbial populations. Kang and Kondo [15] ascribed the rapid reduction of BPA to an increase in bacterial counts in water samples, while Klecka [28] found no relationship between BPA removal and bacterial counts. The different outcomes may have resulted from different bacterial community compositions [16]. In this study, indigenous microorganisms may include bacteria, algae and fungi, indicating more complicated situations in response to nutrient addition. Accordingly, further research should be conducted to elucidate the mechanisms responsible for the increased BPA degradation with nutrient addition. Although nutrient addition increased BPA removal by indigenous microorganisms, the removal rate was still low (<15%) at 12 days when the initial BPA concentration was 5.0 mg L^−1^ under both nutrient levels. These results indicated that indigenous microorganisms in Donghu lake water had limited ability in BPA removal, and that other remediation methods like phytoremediation should be considered.

### 4.2. Aseptic Seedlings of C. demersum and M. spicatum Greatly Enhanced BPA Removal from Lake Water

Bisphenol A in lake water was rapidly removed after the addition of aseptic seedlings of both species, with levels decreasing to below the detection limit in 3 days when the initial BPA concentration was 0.5 mg L^−1^. Greater biomass density facilitated the BPA removal process, with 100% removal being obtained in 3 days when the biomass density was 10 mg L^−1^ and the initial BPA concentration was 5.0 mg L^−1^. The BPA dissipation efficiency calculated based on the BPA removal per gram of both plant species tested in this study was about 0.17 mg g^−1^ d^−1^, which was much larger than that of other plant species in previous studies, such as *Portulaca oleracea* (0.13 mg g^−1^ d^−1^) [29], *Salvia nemorosa* (0.10 mg g^−1^ d^−1^) [30] and *Azolla filiculoides* (0.06 mg g^−1^ d^−1^) [31]. The high efficiency may have resulted from the characteristics of submerged macrophytes, whose stems and leaves are completely immersed in waters subjected to pollutants, resulting in bioaccumulation and biotransformation [20], especially for hydrophobic organic pollutions such as BPA [32]. Moreover, the addition of submerged plants may improve the development of indigenous microbial populations via additional nutrients supplied from plant secretions [33], which may also favor the BPA removal process. Overall, the enhanced BPA removal process was substantially confirmed in lake water by addition of plant seedlings in this research.

Biodegradation plays a key role in the BPA removal process by plants because there is little biological accumulation [14,34]. Enzymes such as peroxidase and polyphenol oxidase are believed to be involved in the biodegradation of BPA by plants [30,35]. However, different metabolic pathways may be taken by different plants in BPA degradation. Further research should be conducted to elucidate the mechanisms involved. Additionally, aseptic seedlings showed better BPA removal ability than naturally collected seedlings of the same species (data were shown in supporting materials), suggesting that aseptic seedlings collected from in vitro culture will be preferred for BPA phytoremediation programs. Enzymes such as peroxidase were more active in aseptic seedlings, indicating that they may be responsible for the observed results (data were shown in supporting materials).

In recent years, the endocrine activity of metabolites has been paid more and more attention to for pollutants such as BPA. According to our research, BPA was mostly degraded by submerged plants in lake water, suggesting the additional concern on the endocrine activity of metabolites was needed. The BPA polluted water after treating with *C. demersum* was then used to test the endocrine activity with zebra fish, and no significant influence was found on the vitellogenin level, indicating lower or no activity (data were shown in supporting materials). Skledar and Mašič (2016) reported the endocrine activity of BPA and its metabolites like 4-methyl-2,4-bis(p-hydroxyphenyl)pent-1-ene (MBP), bisphenol A glucuronide (BPA-G), isopropenylphenol and so on [36]. Some showed no endocrine activity (like BPA-G), some is comparable to BPA (like isopropenylphenol), while others showed higher activity than BPA (like MBP). Further research was needed.

### 4.3. C. demersum Was Preferred in BPA Phytoremediation

Both plant species investigated in this study had great ability in BPA removal. However, comparison of the species revealed that *C. demersum* always had better performance for BPA removal than *M. spicatum*, suggesting that the former species should be more suitable for phytoremediation of BPA polluted waters. This difference may be caused by differences in their metabolic pathways, which should be investigated in further research. According to early reports, different metabolites of BPA can be produced by different plant species, like BPA-mono-O-β-D-glucopyranoside by *Nicotiana tabacum* and *Ipomoea aquatica* [34,37], 4-isopropenylphenol by *Armoracia rusticana* [38] and BPA-OH by *Portulaca oleracea* [39], indicating their different metabolic pathways of BPA. Besides, morphological and/or anatomy structure of plants can also affect the absorption of pollutants [40], resulting in different BPA removal ability.

Both plant species showed tolerance to BPA pollution based on the fact that plant growth was not significantly inhibited by increases in initial BPA concentration until it reached 10 mg L^−1^, at which point the growth of *M. spicatum* was severely inhibited. Therefore, both species are good candidates for BPA phytoremediation considering the low concentration of BPA in natural lake and river waters, although *C. demersum* is preferred.

## 5. Conclusions

Indigenous microorganisms in Donghu lake waters have limited ability to remove BPA, especially at higher initial concentrations, suggesting that a supplementary method is needed for remediation. Seedlings of *C. demersum* and *M. spicatum* from in vitro culture were found to be good candidates for phytoremediation activities because of their extraordinary performance at BPA removal, especially the former species. A higher biomass density of 10 g L^−1^ was favorable for the process, as indicated by nearly 100% BPA removal in 3 days. However, nutrient conditions had little effect on BPA removal by plants plus indigenous microorganisms. Additionally, *C. demersum* was more tolerant to BPA pollution than *M. spicatum*, indicating that the former species is preferred for BPA phytoremediation.

## Figures and Tables

**Table 1:** Results of two-way ANOVAs (*F* value) evaluating the effects of initial Bisphenol A (BPA) concentration (mg L^−1^) and biota treatments on BPA removal rates under different nutrient conditions for different experimental times (days).

Nutrient Conditions	Experimental Time	BPA Initial Concentration	Biota Treatment	BPA initial Concentration × Biota Treatment
Original condition	3	5.27 *	216.99 **	1.09
	6	10.73 **	422.83 **	0.09
	9	13.75 **	612.51**	0.37
	12	14.75 **	864.96 **	1.17
Nutrient addition condition	3	10.24 **	215.36 **	0.85
	6	43.55 **	226.59 **	19.97 **
	9	124.05 **	352.14 **	72.94 **
	12	190.93 **	544.61 **	113.16 **

* and ** indicate significance at 0.05 and 0.01 significance level.

**Table 2:** Results of three-way ANOVAs (*F* value) evaluating the effect of species type, initial BPA concentration (mg L^−1^) and biomass density (g L^−1^) on BPA removal rate under different nutrient conditions during different experimental times (days).

Nutrient Condition	Experimental Time	Species Type	BPA Initial Concentration	Biomass Density	Species Type × BPA Initial Concentration	Species Type × Biomass Density	BPA Initial Concentration × Biomass Density	Species Type × BPA Initial Concentration × Biomass Density
Original nutrient condition	3	55.49 **	274.56 **	177.53 **	55.49 **	17.67 **	177.53 **	17.67 **
	6	75.93 **	259.22 **	176.47 **	75.93 **	34.78 **	176.47 **	34.78 **
	9	56.43 **	164.25 **	137.32 **	56.43 **	41.14 **	137.32 **	41.14 **
	12	136.78 **	311.06 **	281.59 **	136.78 **	117.48 **	281.59 **	117.48 **
Nutrient addition condition	3	223.35 **	1860.43 **	1860.43 **	223.35 **	223.35 **	1860.43 **	223.35 **
	6	200.49 **	750.98 **	750.98 **	200.49 **	200.49 **	750.98 **	200.49 **
	9	109.38 **	215.51 **	215.57 **	111.21 **	111.17 **	213.02 **	113.01 **
	12	25.02 **	41.23 **	41.23 **	25.02 **	25.02 **	41.23 **	25.02 **

** indicates significance at 0.01 level.

**Table 3:** Results of two-way ANOVAs (F value) evaluating the effects of initial BPA concentration (mg/L) and biomass density (g/L) on relative growth rate of *Ceratophyllum demersum* and *Myriophyllum spicatum* under different nutrient conditions.

Nutrient Condition	Plant Species	BPA Initial Concentration	Biomass Density	BPA Initial Concentration × Biomass Density
Original nutrient condition	*C. demersum*	2.004	117.936 **	26.050 **
	*M. spicatum*	35.122 **	188.311 **	6.906 *
Nutrient addition condition	*C. demersum*	29.584 **	113.300 **	1.412
	*M. spicatum*	112.590 **	225.467 **	9.205 *

* and ** indicate significance at 0.05 and 0.01 level, respectively.

## Data Availability

The data presented in this study are available on request from the corresponding author.

## Supplementary Materials

The following are available online at https://www.mdpi.com/1660-4601/18/2/810/s1, Figure S1: the BPA removal rates of wild plant and aseptic seedlings of Ceratophyllum demersum and Myriophyllum spicatum in 24 h. Figure S2: the peroxidase activities of wild plant and aseptic seedlings of Ceratophyllum demersum and Myriophyllum spicatum. Figure S3: The VTG (mg/L) concentration of plasma in zebra fish in treated water with (represent as Metabolite) and unpolluted water (represent as CK).
