# Supplementary material for: Enhanced Phytoremediation of Bisphenol A in Polluted Lake Water by Seedlings of Ceratophyllum demersum and Myriophyllum spicatum from In Vitro Culture
Source: Int J Environ Res Public Health. 2021 Jan 19;18(2):810. doi: 10.3390/ijerph18020810 (PMC7832867; doi:10.3390/ijerph18020810)
Supplement: Supplementary file 1 [file ijerph-18-00810-s001.pdf]

Supporting data

The aseptic seedlings of *Ceratophyllum demersum* showed a better BPA removal rate than that of wild plant (Figure S1), which was about twice as high in the aseptic seedlings as in the wild macrophyte samples. There is a similar result in removing BPA by *Myriophyllum spicatum.* These results indicated that the aseptic seedlings of macrophyte had a better BPA removal ability than that of wild plant.

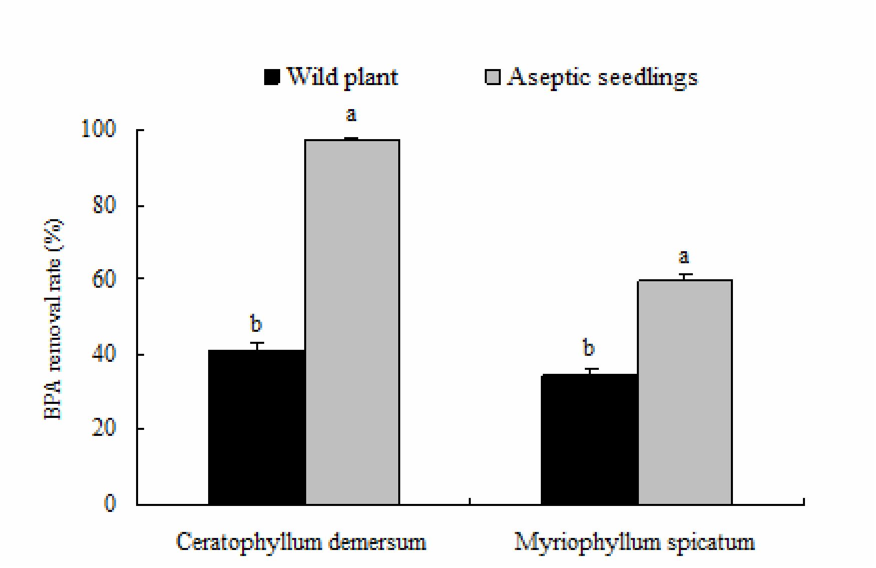

Figure S1 the BPA removal rates of wild plant and aseptic seedlings of *Ceratophyllum demersum* and *Myriophyllum spicatum* in 24h. Data are presented as the Mean±S.D. (n=3), different letter in the same group means significant difference at 0.05 level

Compared the peroxidase (POD) activities of aseptic seedlings and wild plant of *Ceratophyllum demersum* (Figure S2), the POD activity of aseptic seedlings was 44000 U/min/g, which was about two times than that of wild plant. The similar result was found in that of *Myriophyllum spicatum.*

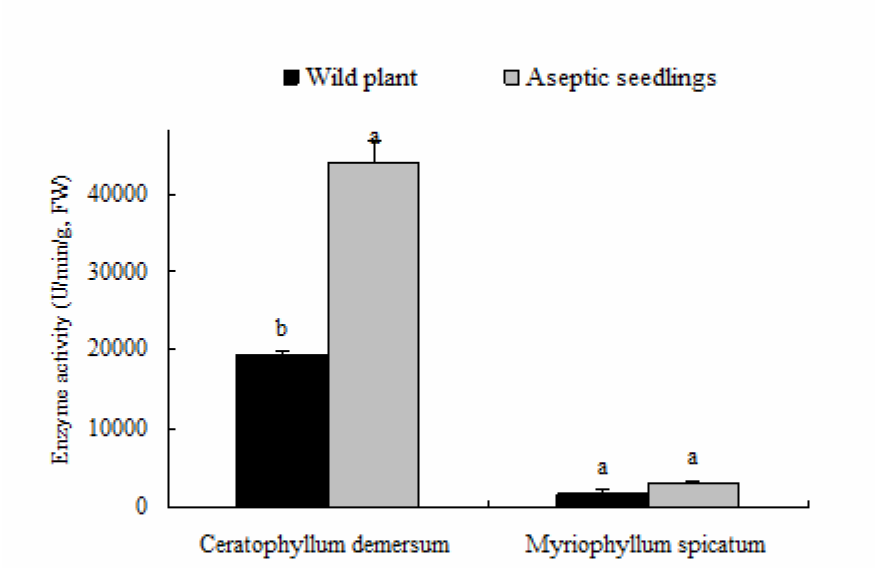

Figure S2 the peroxidase activities of wild plant and aseptic seedlings of *Ceratophyllum demersum* and *Myriophyllum spicatum*. Data are presented as the Mean±S.D. (n=3), different letter in the same group means significant difference at 0.05 level

Comparing the vitellogenin level of zebra fish in treated water with *C. demersum* (5 mg L^{-1} initial BPA concentration) and unpolluted water (0 mg L-1 BPA as Control), no significance was found **(Figure S3)**.

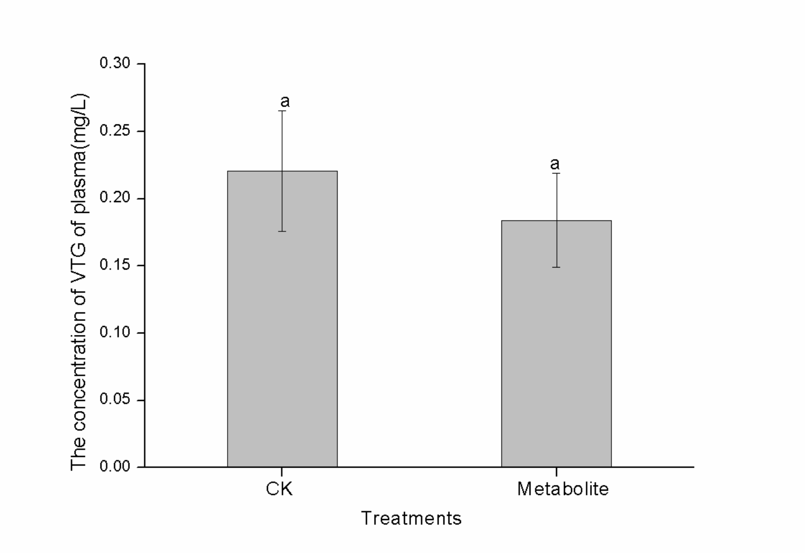

Figure S3 The VTG (mg/L) concentration of plasma in zebra fish in treated water with (represent as Metabolite) and unpolluted water (represent as CK)
